# Supplementary material for: Association between Psoriasis and Renal Functions: An Integration Study of Observational Study and Mendelian Randomization
Source: Biomedicines. 2024 Jan 22;12(1):249. doi: 10.3390/biomedicines12010249 (PMC10813483; doi:10.3390/biomedicines12010249)
Supplement: Supplementary file 1 [file biomedicines-12-00249-s001.zip › Supplementary File.pdf]

Association between psoriasis and renal functions: an integration study of observational study and Mendelian Randomization

Yuxuan Tan[a,#], Zhizhuo Huang[a,b,#], Haiyin Li[a], Huojie Yao[a], Yingyin Fu[a], Xiaomei Wu[a], Chuhang Lin[a], Zhengtian Lai[a], Guang Yang[b,*], Chunxia Jing[a,*]

AFFILIATIONS

aDepartment of Epidemiology, School of Medicine, Jinan University, No.601 Huangpu Ave West, Guangzhou 510632, Guangdong, P. R. China

bDepartment of Pathogen Biology, School of Medicine, Jinan University, No.601 Huangpu Ave West, Guangzhou 510632, Guangdong, P. R. China

#These authors contributed equally.

CORRESPONDING AUTHOR

*Correspondence:

Chunxia Jing (C-X.J): jcxphd@gmail.com

Guang Yang (G.Y.): tyangguang@email.jnu.edu.cn

Supplementary file contents

Supplementary Table

Table S1 Data sources for summary GWAS data.

Table S2 Characteristics of summary-level GWAS datasets of competing risks for MVMR study.

Table S3 Distribution of three continuous kidney functions, NHANES 2003–2006 and 2009–2014.

Table S4 Detailed results of sensitivity analyzes of epidemiological observational studies.

Table S5 Genetic variants of psoriasis used as potential instruments in the forward MR analyses.

Table S6 Genetic variants used as potential instruments in the reverse Mendelian randomization analyses.

Table S7 Multi MR methods results for kidney function on psoriasis, reverse direction.

Table S8 The heterogeneity test calculated based on IVW method.

Table S9 The results of MR-Egger intercept for pleiotropy test.

Table S10 CAUSE-based MR analysis for psoriasis on four kidney functions.

Table S11 Genetic variants used as potential instruments in the MVMR analyses

Supplementary Figure

Fig. S1 Directed acyclic graph (DAG) of covariates in the association between psoriasis and kidney function.

Fig. S2 Flow chart for the MR framework analysis.

Fig. S3 The subgroup analysis results for psoriasis on eGFR (Fig. S3A), UACR (Fig. S3B), BUN (Fig. S3C), and CKD (Fig. S3D).

Fig. S4 LDSC results for psoriasis and four kidney functions.

Fig. S5 The forward associations of psoriasis on renal functions in each instrumental variable.

Fig. S6 The funnel plot of the effect between psoriasis and four renal functions.

Fig. S7 Causal Analysis Using Summary Effect estimates (CAUSE) for psoriasis on four renal functions.

Fig. S8 The leave-one-out plot of the effect of psoriasis on four renal functions.

Supplementary Table

Table S1 Data sources for summary GWAS data.

Data	Data source	PMID	Population ancestry	Sample size	Case	Control	Exposure definition	Adjustments
**Psoriasis**								
Psoriasis	FINNGEN Consortium	36653562	European	252323	5621	246702	ICD10 - L40	Age, sex, ten genetic principal components, and genotyping batch
**Kidney Fuction**								
Log-transformed eGFR based on creatinine levels (eGFR)	Chronic kidney disease genetics consortium meta-analysis (n = 42 studies)	34272381	European	567460	–	–	Change in log eGFR (mL min−1 per 1.73 m2) per copy increment of effect allele	
Blood urea nitrogen (BUN)	Chronic kidney disease genetics consortium meta-analysis (n = 24 studies)	34272381	European	243029	–	–	Change in BUN (mg/dL) per copy increment of effect allele	Sex and age in all included studies, with some studies in the meta-analysis further adjusting for study site, relatedness, and principal components of genetic ancestry
Urine albumin-creatinine ratio (UACR)	Chronic kidney disease genetics consortium meta-analysis (n = 18 studies)	31511532	European	547361	–	–	Change in UACR (mg/g) per copy increment of effect allele	
Chronic kidney disease (CKD)	Chronic kidney disease genetics consortium meta-analysis (n = 23 studies)	31152163	European	480698	41395	439303	Log odds ratio for CKD per copy increment of effect allele	

Note: PMID, PubMed Unique Identifier. Continuous variables do not demonstrate the numbers of cases and controls.

Table S2 Characteristics of summary-level GWAS datasets of competing risks for MVMR study.

Summary-level GWAS data	Souce/Consortium	PMID[a]	Definition	Ancestry	Case	Controls	Sample size
Lifestyle							
Smoking	UKBB	31427789	self-reported history of drinking, ever drink	European	235098	149918	385016
Drinking	UKBB	31427789	self-reported history of smoking, ever smoke	European	12522	373560	386082
Physical activity (PA)	UKBB	29899525	self-reported history of vigorous PA, activities that make participants sweat or breathe hard such as fast cycling, aerobics, heavy lifting	European	98060	162995	261055
Competing diseases							
Obesity	FinnGen	-	defined by ICD-10, coded E65-E66	European	15517	293547	309064
Type 2 diabetes	UKBB	31427789	defined by ICD-10, coded E11	European	16673	228217	244890

Note: a. PubMed Unique Identifier, it is the document number of life science, medicine and other fields included in PubMed search engine.

Table S3 Distribution of three continuous kidney functions, NHANES 2003–2006 and 2009–2014.

Kidney function	Percentile					GM (95%CI)
	5%	25%	50%	75%	95%	
eGFR	58.65	84.99	99.57	111.58	126.22	93.83 (93.45, 94.21)
UACR	2.50	4.19	6.49	12.13	75.81	8.39 (8.26, 8.52)
BUN	6.00	9.00	12.00	15.00	21.00	11.78 (11.72, 11.84)

Note: GM, Geometric Means; eGFR, estimated glomerular filtration rate based on creatinine levels; UACR, urine albumin-creatinine ratio; BUN, blood urea nitrogen.

Table S4 Detailed results of sensitivity analyzes of epidemiological observational studies.

Kidney functions	Total participants	Estimate	SE	Lower 95% CI	Upper 95%CI	P-value
**Fitted with complete dataset generated by multivariate imputations[a]**						
eGFR	19238	-2.437	1.094	-4.583	-0.292	**0.030**
UACR	20070	-0.009	0.046	-0.100	0.081	0.839
BUN	19237	0.009	0.021	-0.032	0.049	0.671
CKD	20244	-0.089	0.149	-0.381	0.204	0.555
**Further adjusted for SII[b]**						
eGFR	13,773	-2.925	1.279	-5.471	-0.379	**0.025**
UACR	13,789	-0.018	0.057	-0.132	0.096	0.755
BUN	13,773	0.025	0.020	-0.016	0.065	0.227
CKD	13,845	0.033	0.199	-0.126	0.192	0.681
**Excluding participants with CKD, diabetes, hypertension, and hyperlipidemia[c]**						
eGFR	6,139	-3.361	1.363	-6.076	-0.647	**0.016**
UACR	6,150	0.031	0.071	-0.110	0.173	0.661
BUN	6,139	-0.013	0.034	-0.081	0.055	0.708
**Remove two NHANES cycles (NHANES 2011-2012, 2013-2014)**						
eGFR	7,178	-3.183	1.414	-6.030	-0.337	**0.029**
UACR	7,186	0.041	0.089	-0.138	0.220	0.650
BUN	7,178	0.033	0.024	-0.016	0.081	0.180
CKD	7,208	0.165	0.418	-0.085	0.415	0.191

Note: a. We imputed observations for all covariates, for which ≤ 10 % of the total observations were missing. Ten imputed datasets with chained equations using a *Mice* package in R were created and fitted the GLM. b. SII was defined as platelet number × peripheral neutrophil number / lymphocyte. Data of blood count can be found in NHANES laboratory data. c. Hyperlipidemia was defined using blood pressure & cholesterol questionnaire "Doctor told you high cholesterol level". Only continuous kidney function variables were investigated, as participants with CKD were excluded. SII, systemic immune-inflammatory index; eGFR, estimated glomerular filtration rate based on creatinine levels; UACR, urine albumin-creatinine ratio; BUN, blood urea nitrogen.

Table S5 Genetic variants of psoriasis used as potential instruments in the forward MR analyses.

Chrom	Positon	SNV	Effect allele	Other allele	log(OR)	*P*-value	SE	EAF
1	24968116	rs7542079	C	T	0.1074	6.05E-10	0.017353	0.562152
2	60890978	rs12713428	C	A	0.139874	7.16E-13	0.019491	0.248465
2	195206980	rs77509633	C	T	-0.33206	1.93E-08	0.059107	0.026182
5	159422483	rs10866712	C	T	-0.17939	1.40E-24	0.017529	0.643231
5	151098757	rs17728338	A	G	0.240034	8.21E-15	0.030915	0.073921
5	159253359	rs6886974	T	A	0.326401	9.19E-12	0.047869	0.027547
5	132660977	rs847	C	T	0.105106	6.00E-09	0.018069	0.635234
6	24958486	rs115086705	T	C	0.240348	3.63E-08	0.043636	0.03468
6	25898470	rs115941737	G	T	0.297957	4.66E-08	0.054531	0.021081
6	31385041	rs118061248	G	A	0.422428	2.94E-31	0.036326	0.045858
6	33596519	rs12206050	T	A	0.117638	8.56E-11	0.018125	0.320433
6	31299200	rs13210419	A	G	0.808407	2.30E-177	0.028469	0.059032
6	31021066	rs137936979	A	G	-0.15219	1.12E-10	0.023593	0.17005
6	27666029	rs141439735	A	C	0.412388	4.17E-09	0.070165	0.011764
6	26619220	rs142948774	G	A	0.418987	4.42E-24	0.041395	0.034115
6	29516191	rs1592410	T	C	-0.12398	3.63E-13	0.017057	0.527712
6	29949027	rs1655901	C	T	0.272367	6.01E-58	0.016973	0.471274
6	111592059	rs33980500	T	C	0.216632	2.94E-12	0.031034	0.072629
6	33011220	rs3763342	T	C	0.225834	9.22E-19	0.025535	0.112524
6	137895651	rs674451	C	T	0.123522	4.06E-12	0.017811	0.343858
6	33028086	rs6933319	T	C	0.378728	4.06E-22	0.039167	0.039223
6	28199005	rs73400551	C	T	0.210142	2.88E-10	0.033329	0.063066
6	30793709	rs74787141	C	T	0.3953	1.23E-44	0.028201	0.078853
6	31294950	rs76343304	T	C	-0.2348	2.84E-11	0.035285	0.072431
7	37342861	rs60600003	G	T	0.166098	9.02E-10	0.027114	0.10187
7	5442506	rs62443225	A	G	0.189129	1.08E-09	0.031018	0.079174
14	35402011	rs8904	A	G	-0.12665	8.89E-13	0.017721	0.388033
16	11251046	rs2021511	T	C	-0.12208	7.14E-10	0.019809	0.265958
17	27797882	rs28998802	A	G	0.161415	4.87E-14	0.021421	0.184892
20	49907751	rs636987	C	A	-0.09818	2.22E-08	0.017551	0.617959

Table S6 Genetic variants used as potential instruments in the reverse Mendelian randomization analyses.

(Table S6 was presented in Excel as an independent file)

Table S7 Multi MR methods results for kidney function on psoriasis, reverse direction.

Exposure	MR methods	Estimate	SE	Lower	Upper	*P*-value
eGFR	MR-IVW	-0.4848	0.6889	-1.8351	0.8655	0.482
eGFR	Weighted median	-1.2618	1.0395	-3.2992	0.7756	0.225
eGFR	Simple mode	-4.5165	3.4416	-11.2620	2.2290	0.191
eGFR	MR-Egger	-0.2665	1.6717	-3.5430	3.0100	0.873
eGFR	MR-RAPs	-0.4419	0.7206	-1.8543	0.9705	0.540
UACR	MR-IVW	0.1958	0.2640	-0.3217	0.7133	0.458
UACR	Weighted median	-0.0868	0.3888	-0.8488	0.6752	0.823
UACR	Simple mode	-0.1356	0.8274	-1.7573	1.4861	0.870
UACR	MR-Egger	-0.4961	0.7625	-1.9906	0.9984	0.518
UACR	MR-RAPs	0.2005	0.2757	-0.3399	0.7409	0.467
BUN	MR-IVW	0.7113	0.5190	-0.3059	1.7285	0.171
BUN	Weighted median	0.4920	0.6806	-0.8420	1.8260	0.470
BUN	Simple mode	1.1235	1.4063	-1.6328	3.8798	0.427
BUN	MR-Egger	1.1607	1.3308	-1.4477	3.7691	0.386
BUN	MR-RAPs	0.6175	0.5377	-0.4364	1.6714	0.251
CKD	MR-IVW	0.1504	0.0791	-0.0046	0.3053	0.061
CKD	Weighted median	0.1234	0.1221	-0.1159	0.3627	0.312
CKD	Simple mode	0.1518	0.2549	-0.3478	0.6514	0.558
CKD	MR-Egger	-0.1566	0.1976	-0.5439	0.2307	0.437
CKD	MR-RAPs	0.1965	0.0838	0.0323	0.3607	0.019

Note: eGFR, estimated glomerular filtration rate based on creatinine levels; UACR, urine albumin-creatinine ratio; BUN, blood urea nitrogen.

Table S8 The heterogeneity test calculated based on IVW method.

Exposure	Cochran's Q	Degree of freedom (DF)	*P*-value
**Psoriasis - Renal Function (Forward)**			
eGFR	79.225	30	**< 0.001**
UACR	53.605	30	**0.007**
BUN	35.468	30	0.265
CKD	40.863	30	0.111

Note: The *P*-value < 0.05 for Cochran's Q statistic indicates significant heterogeneity across instrument SNV effects.

Table S9 The results of MR-Egger intercept for pleiotropy test.

Exposure	Egger intercept	SE	*P*-value
**Psoriasis - Renal Function (Forward direction)**			
eGFR	8.67E-05	0.0003	0.7640
BUN	-6.80E-04	0.0005	0.1519
UACR	9.27E-04	0.0013	0.4911
CKD	-7.44E-03	0.0054	0.1771

Table S10 CAUSE-based MR analysis for psoriasis on four kidney functions.

Renal function	Model 1	Model 2	ΔELPD (SE)	γ (95% CI)	η (95% CI)	q (95% CI)	*P*-value
$eGFR_{creat}$							
	Null	Sharing	0.34 (0.33)	-	0 (-0.05, 0.03)	0.04 (0, 0.24)	0.850
	Null	Causal	-0.14 (1.70)	0 (0, 0)	0 (-0.05, 0.03)	0.03 (0, 0.23)	0.470
	Sharing	Causal	-0.48 (1.50)	-	-	-	0.380
UACR							
	Null	Sharing	0.34 (0.08)	-	0 (-0.08, 0.08)	0.04 (0, 0.25)	1.000
	Null	Causal	0.52 (0.78)	0 (-0.01, 0)	0 (-0.07, 0.08)	0.04 (0, 0.25)	0.750
	Sharing	Causal	0.17 (0.72)	-	-	-	0.590
BUN							
	Null	Sharing	0.30 (0.42)	-	0.01 (-0.04, 0.06)	0.04 (0, 0.23)	0.760
	Null	Causal	-0.13 (1.10)	0 (0, 0.01)	0.01 (-0.04, 0.06)	0.04 (0, 0.23)	0.450
	Sharing	Causal	-0.43 (0.79)	-	-	-	0.290
CKD							
	Null	Sharing	0.34 (0.12)	-	0 (-0.40, 0.50)	0.04 (0, 0.24)	1.000
	Null	Causal	1.50 (0.36)	0 (-0.04, 0.04)	0 (-0.39, 0.49)	0.05 (0, 0.28)	1.000
	Sharing	Causal	1.10 (0.31)	-	-	-	1.000

Note: Model 1 and model 2 refer to the models being compared (null, sharing, or causal). Model fit is measured by Δ Expected Log Pointwise Posterior Density (ΔELPD); ΔELPD: Estimated difference in ELPD.

Table S11 Genetic variants used as potential instruments in the MVMR analyses.

(Table S11 was presented in Excel as an independent file)

Supplementary Figure

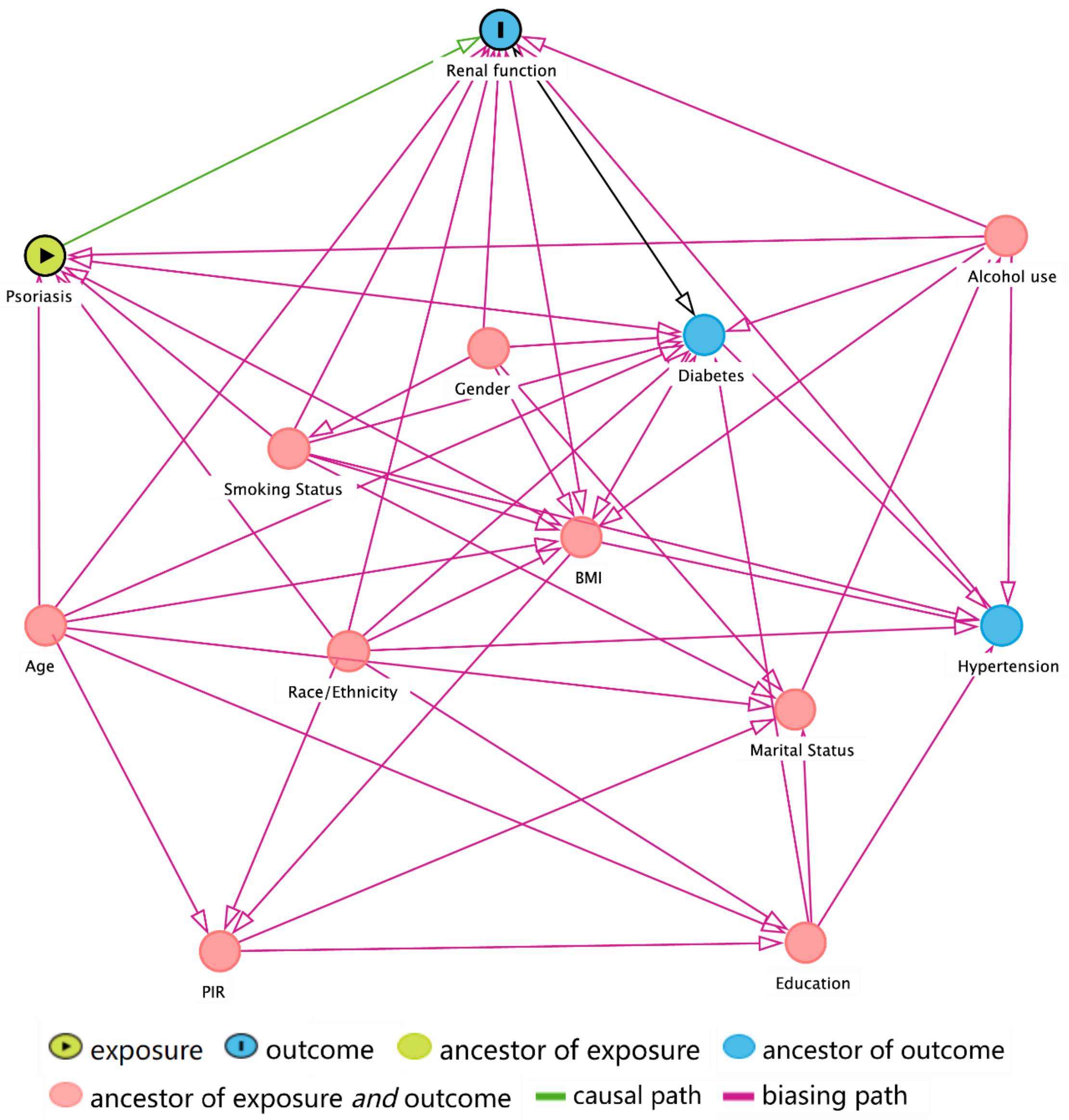

Fig. S1 Directed acyclic graph (DAG) of covariates in the association between psoriasis and kidney function.

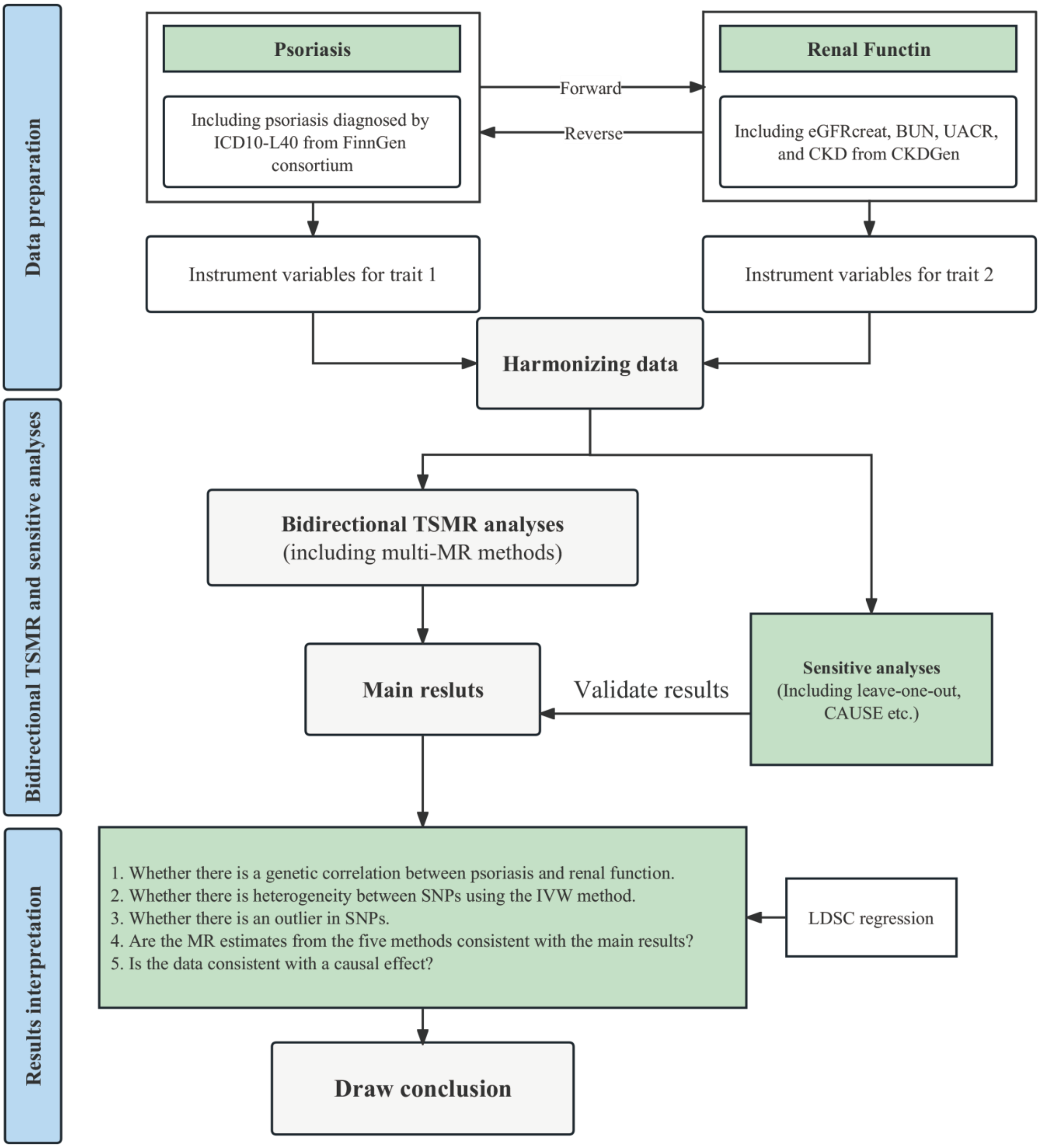

Fig. S2 Flow chart for the MR framework analysis. The MR method depends on three key assumptions: 1. the exposure should directly link to the genetic variations used as instrumental variables (IVs); 2. there are no associations between IVs and any confounding variables; 3. the IVs impact outcome only through the exposure.

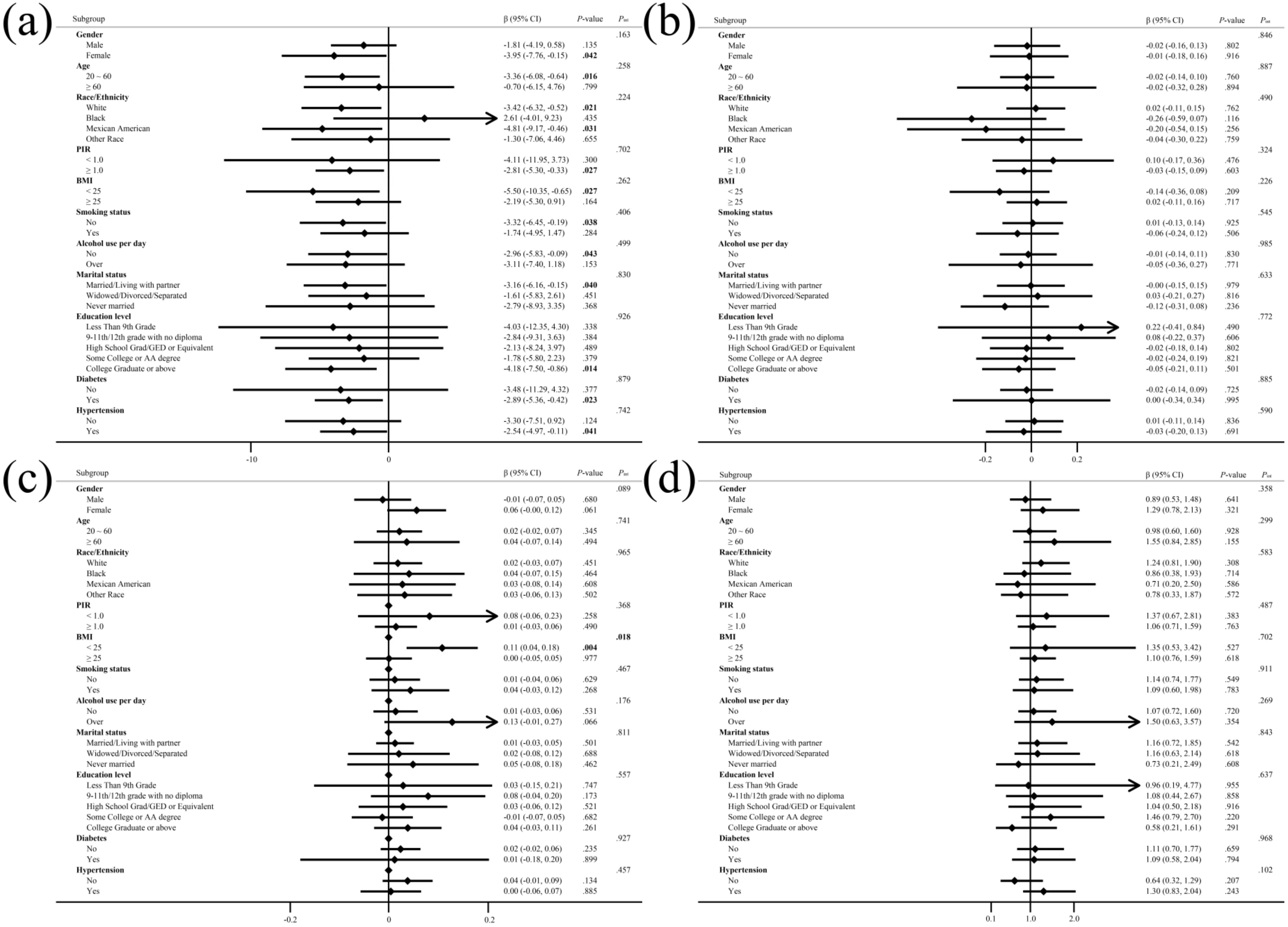
(a)
Subgroup
β (95% CI)
P-value
Pint
Gender .163
Male -1.81 (-4.19, 0.58) .135
Female -3.95 (-7.76, -0.15) .042
Age .258
20 ~ 60 -3.36 (-6.08, -0.64) .016
≥ 60 -0.70 (-6.15, 4.76) .799
Race/Ethnicity .224
White -3.42 (-6.32, -0.52) .021
Black 2.61 (-4.01, 9.23) .435
Mexican American -4.81 (-9.17, -0.46) .031
Other Race -1.30 (-7.06, 4.46) .655
PIR .702
< 1.0 -4.11 (-11.95, 3.73) .300
≥ 1.0 -2.81 (-5.30, -0.33) .027
BMI .262
< 25 -5.50 (-10.35, -0.65) .027
≥ 25 -2.19 (-5.30, 0.91) .164
Smoking status .406
No -3.32 (-6.45, -0.19) .038
Yes -1.74 (-4.95, 1.47) .284
Alcohol use per day .499
No -2.96 (-5.83, -0.09) .043
Over -3.11 (-7.40, 1.18) .153
Marital status .830
Married/Living with partner -3.16 (-6.16, -0.15) .040
Widowed/Divorced/Separated -1.61 (-5.83, 2.61) .451
Never married -2.79 (-8.93, 3.35) .368
Education level .926
Less Than 9th Grade -4.03 (-12.35, 4.30) .338
9-11th/12th grade with no diploma -2.84 (-9.31, 3.63) .384
High School Grad/GED or Equivalent -2.13 (-8.24, 3.97) .489
Some College or AA degree -1.78 (-5.80, 2.23) .379
College Graduate or above -4.18 (-7.50, -0.86) .014
Diabetes .879
No -3.48 (-11.29, 4.32) .377
Yes -2.89 (-5.36, -0.42) .023
Hypertension .742
No -3.30 (-7.51, 0.92) .124
Yes -2.54 (-4.97, -0.11) .041
-10
0
(b)
Subgroup
β (95% CI)
P-value
Pint
Gender .846
Male -0.02 (-0.16, 0.13) .802
Female -0.01 (-0.18, 0.16) .916
Age .887
20 ~ 60 -0.02 (-0.14, 0.10) .760
≥ 60 -0.02 (-0.32, 0.28) .894
Race/Ethnicity .490
White 0.02 (-0.11, 0.15) .762
Black -0.26 (-0.59, 0.07) .116
Mexican American -0.20 (-0.54, 0.15) .256
Other Race -0.04 (-0.30, 0.22) .759
PIR .324
< 1.0 0.10 (-0.17, 0.36) .476
≥ 1.0 -0.03 (-0.15, 0.09) .603
BMI .226
< 25 -0.14 (-0.36, 0.08) .209
≥ 25 0.02 (-0.11, 0.16) .717
Smoking status .545
No 0.01 (-0.13, 0.14) .925
Yes -0.06 (-0.24, 0.12) .506
Alcohol use per day .985
No -0.01 (-0.14, 0.11) .830
Over -0.05 (-0.36, 0.27) .771
Marital status .633
Married/Living with partner -0.00 (-0.15, 0.15) .979
Widowed/Divorced/Separated 0.03 (-0.21, 0.27) .816
Never married -0.12 (-0.31, 0.08) .236
Education level .772
Less Than 9th Grade 0.22 (-0.41, 0.84) .490
9-11th/12th grade with no diploma 0.08 (-0.22, 0.37) .606
High School Grad/GED or Equivalent -0.02 (-0.18, 0.14) .802
Some College or AA degree -0.02 (-0.24, 0.19) .821
College Graduate or above -0.05 (-0.21, 0.11) .501
Diabetes .885
No -0.02 (-0.14, 0.09) .725
Yes 0.00 (-0.34, 0.34) .995
Hypertension .590
No 0.01 (-0.11, 0.14) .836
Yes -0.03 (-0.20, 0.13) .691
-0.2
0
0.2
(c)
Subgroup
β (95% CI)
P-value
Pint
Gender .089
Male -0.01 (-0.07, 0.05) .680
Female 0.06 (-0.00, 0.12) .061
Age .741
20 ~ 60 0.02 (-0.02, 0.07) .345
≥ 60 0.04 (-0.07, 0.14) .494
Race/Ethnicity .965
White 0.02 (-0.03, 0.07) .451
Black 0.04 (-0.07, 0.15) .464
Mexican American 0.03 (-0.08, 0.14) .608
Other Race 0.03 (-0.06, 0.13) .502
PIR .368
< 1.0 0.08 (-0.06, 0.23) .258
≥ 1.0 0.01 (-0.03, 0.06) .490
BMI .018
< 25 0.11 (0.04, 0.18) .004
≥ 25 0.00 (-0.05, 0.05) .977
Smoking status .467
No 0.01 (-0.04, 0.06) .629
Yes 0.04 (-0.03, 0.12) .268
Alcohol use per day .176
No 0.01 (-0.03, 0.06) .531
Over 0.13 (-0.01, 0.27) .066
Marital status .811
Married/Living with partner 0.01 (-0.03, 0.05) .501
Widowed/Divorced/Separated 0.02 (-0.08, 0.12) .688
Never married 0.05 (-0.08, 0.18) .462
Education level .557
Less Than 9th Grade 0.03 (-0.15, 0.21) .747
9-11th/12th grade with no diploma 0.08 (-0.04, 0.20) .173
High School Grad/GED or Equivalent 0.03 (-0.06, 0.12) .521
Some College or AA degree -0.01 (-0.07, 0.05) .682
College Graduate or above 0.04 (-0.03, 0.11) .261
Diabetes .927
No 0.02 (-0.02, 0.06) .235
Yes 0.01 (-0.18, 0.20) .899
Hypertension .457
No 0.04 (-0.01, 0.09) .134
Yes 0.00 (-0.06, 0.07) .885
-0.2
0
0.2
(d)
Subgroup
β (95% CI)
P-value
Pint
Gender .358
Male 0.89 (0.53, 1.48) .641
Female 1.29 (0.78, 2.13) .321
Age .299
20 ~ 60 0.98 (0.60, 1.60) .928
≥ 60 1.55 (0.84, 2.85) .155
Race/Ethnicity .583
White 1.24 (0.81, 1.90) .308
Black 0.86 (0.38, 1.93) .714
Mexican American 0.71 (0.20, 2.50) .586
Other Race 0.78 (0.33, 1.87) .572
PIR .487
< 1.0 1.37 (0.67, 2.81) .383
≥ 1.0 1.06 (0.71, 1.59) .763
BMI .702
< 25 1.35 (0.53, 3.42) .527
≥ 25 1.10 (0.76, 1.59) .618
Smoking status .911
No 1.14 (0.74, 1.77) .549
Yes 1.09 (0.60, 1.98) .783
Alcohol use per day .269
No 1.07 (0.72, 1.60) .720
Over 1.50 (0.63, 3.57) .354
Marital status .843
Married/Living with partner 1.16 (0.72, 1.85) .542
Widowed/Divorced/Separated 1.16 (0.63, 2.14) .618
Never married 0.73 (0.21, 2.49) .608
Education level .637
Less Than 9th Grade 0.96 (0.19, 4.77) .955
9-11th/12th grade with no diploma 1.08 (0.44, 2.67) .858
High School Grad/GED or Equivalent 1.04 (0.50, 2.18) .916
Some College or AA degree 1.46 (0.79, 2.70) .220
College Graduate or above 0.58 (0.21, 1.61) .291
Diabetes .968
No 1.11 (0.70, 1.77) .659
Yes 1.09 (0.58, 2.04) .794
Hypertension .102
No 0.64 (0.32, 1.29) .207
Yes 1.30 (0.83, 2.04) .243
0.1
1.0
2.0

Fig. S3 The subgroup analysis results for psoriasis on eGFR (Fig. S3a), UACR (Fig. S3b), BUN (Fig. S3c), and CKD (Fig. S3d). P_{int}, *P*-value for interaction term Wald test; eGFR, estimated glomerular filtration rate based on creatinine levels; UACR, urine albumin-creatinine ratio; BUN, blood urea nitrogen.

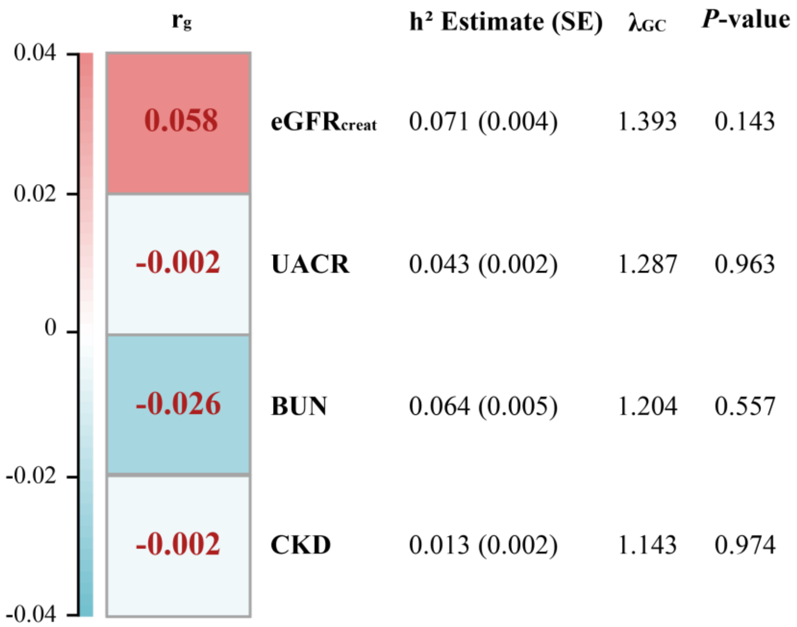

Fig. S4 LDSC results for psoriasis and four kidney functions. Red color indicates positive genetic correlations, and blue color indicates negative genetic correlations.

Note: λ_{GC} indicates the genomic inflation factor, which is calculated as the median χ^2 statistic across SNVs divided by the median χ^2 statistic of the expected χ^2 distribution. r_g, genetic correlation; h^2, estimation of heritability; eGFR, estimated glomerular filtration rate based on creatinine levels; UACR, urine albumin-creatinine ratio; BUN, blood urea nitrogen.

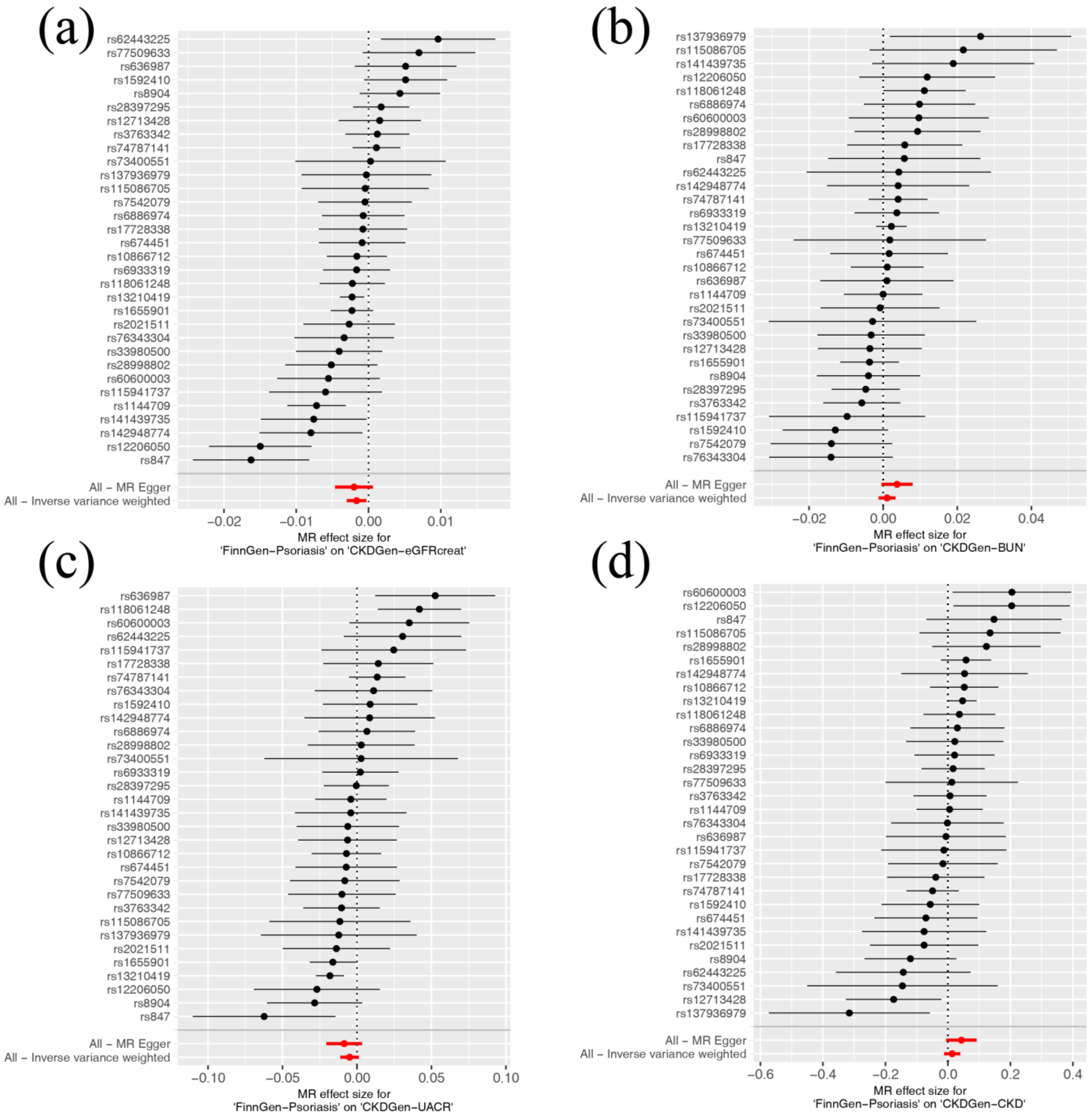

Fig. S5 The forward associations of psoriasis on renal functions in each instrumental variable. Forest plot shows the individual Wald ratios and 95% confidence intervals for each variant used in the primary analysis. Pooled estimates using inverse-variance weighted and MR-Egger are shown in red. The letters represent the kidney functions, A for eGFR, B for BUN, C for UACR, and D for CKD.

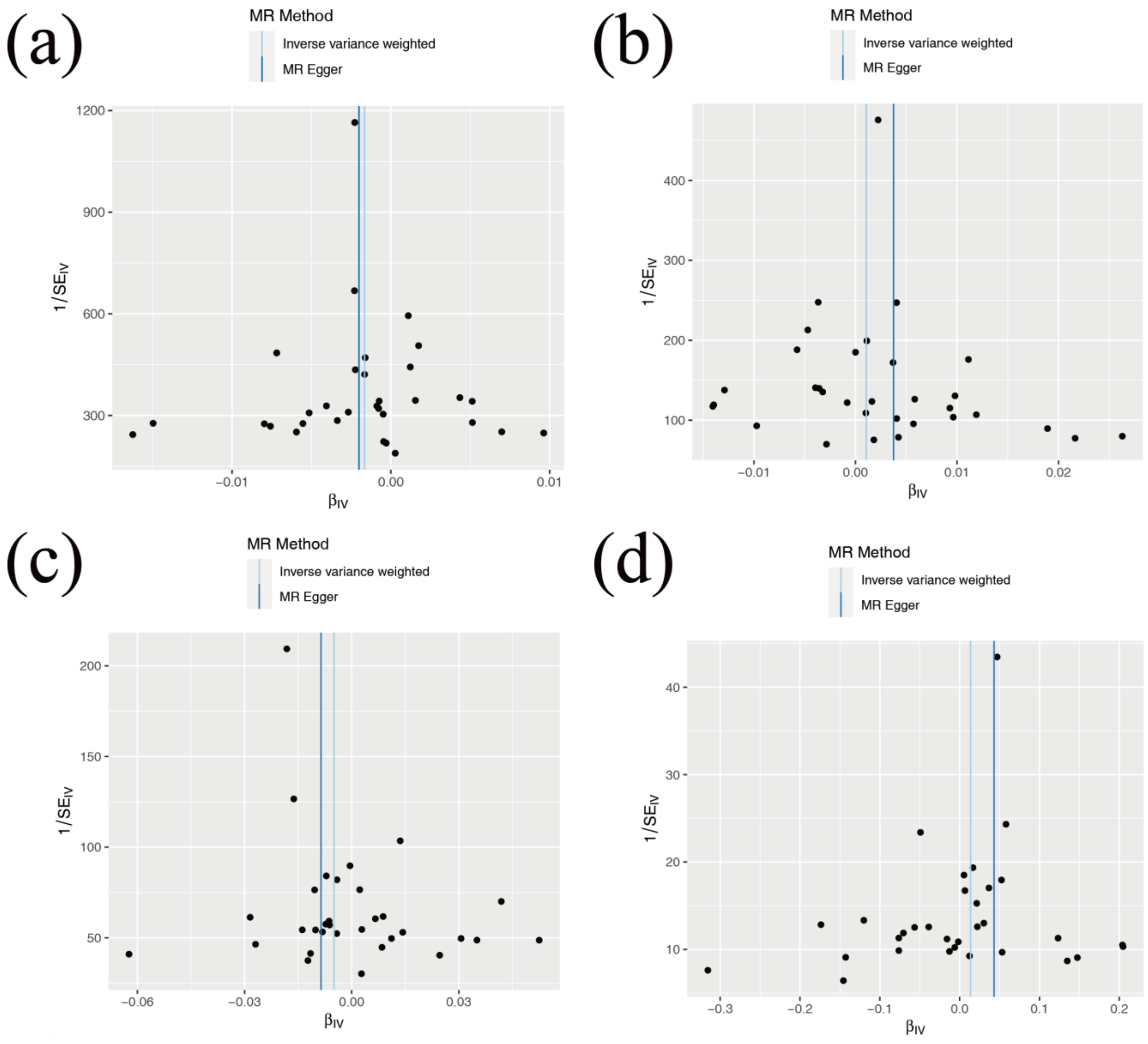

Fig. S6 The funnel plot of the effect between psoriasis and four renal functions. The letters represent the kidney functions, a for eGFR, b for BUN, c for UACR, and d for CKD.

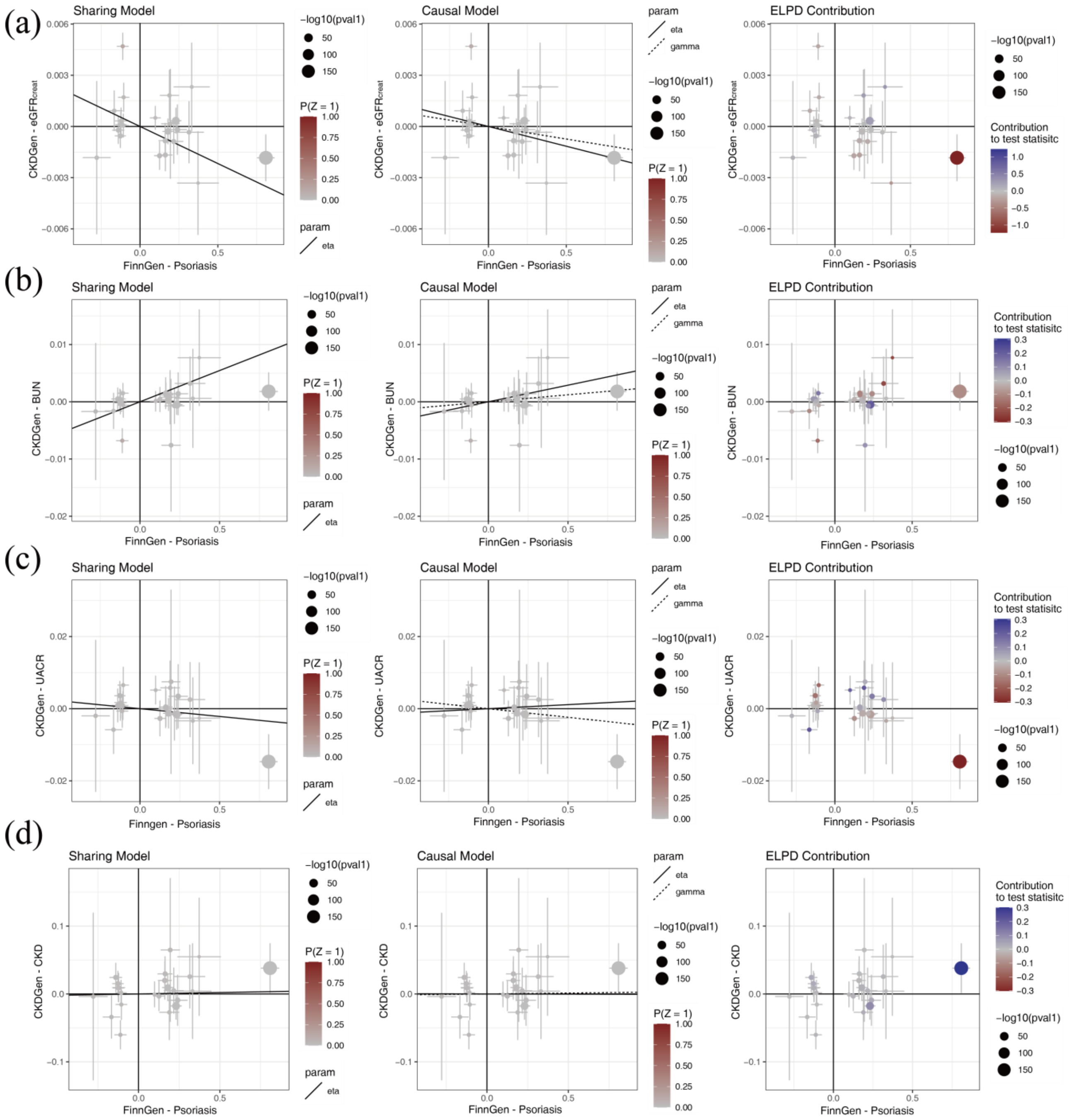

Fig. S7 Causal Analysis Using Summary Effect estimates (CAUSE) for psoriasis on four renal functions. The letters represent the renal functions, a for BUN, b for eGFR, c for UACR, and d for CKD. Neither shared nor causal models appear to fit in comparison to the null model, providing limited evidence for a causal effect of amount of psoriasis on renal functions. The Gamma and Eta in parameter indicate the estimate of causal effect if causal model is correct and estimate of correlated pleiotropy respectively.

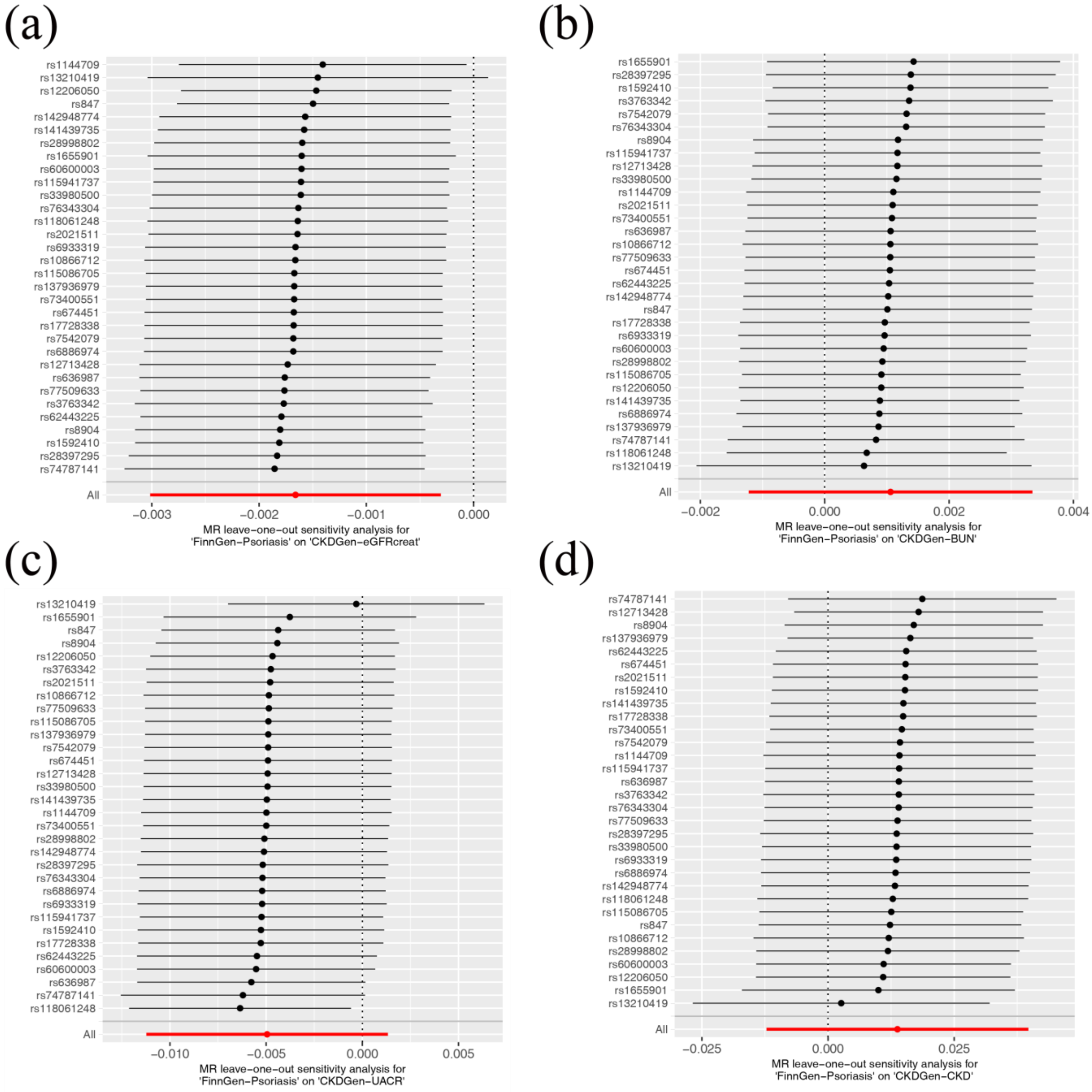

Fig. S8 The leave-one-out plot of the effect of psoriasis on four renal functions. Pooled estimates are shown in red. The letters represent the renal functions, a for eGFR, b for BUN, c for UACR, and d for CKD.
